# *CDK4* loss-of-function mutations cause microcephaly and short stature

**DOI:** 10.1101/gad.352311.124

**Published:** 2025-05-01

**Authors:** Aitana Verdu Schlie, Andrea Leitch, Maria Izabel Arismendi, Colin Stok, Andrea Castro Leal, David A. Parry, Antonio Marcondes Lerario, Margaret E. Harley, Bruna Lucheze, Paula L. Carroll, Kamila I. Musialik, Julia M.T. Auer, Carol-Anne Martin, Lukas Gerasimavicius, Alan J. Quigley, Joya Emilie de Menezes Correia-Deur, Joseph A. Marsh, Martin A.M. Reijns, Anne K. Lampe, Andrew P. Jackson, Alexander A.L. Jorge, Lukas Tamayo-Orrego

**Affiliations:** 1 MRC Human Genetics Unit, Institute of Genetics and Cancer, University of Edinburgh, Edinburgh EH4 2XU, United Kingdom;; 2 Genetic Endocrinology Unit (LIM25), Endocrinology Division, Faculdade de Medicina da Universidade de São Paulo (HC-FMUSP), São Paulo 01246-903, Brazil;; 3 Department of Integrated Health, State University of Para, Santarem 68010-200, Brazil;; 4 Division of Metabolism, Endocrinology, and Diabetes, Department of Internal Medicine, University of Michigan, Ann Arbor, Michigan 48109, USA;; 5 Paediatric Imaging Department, Royal Hospital for Children and Young People, Edinburgh EH16 4TJ, United Kingdom;; 6 South East of Scotland Clinical Genetics Service, Western General Hospital, Edinburgh EH4 2XU, United Kingdom

**Keywords:** cell cycle, centrosome, cyclin-dependent kinase, microcephalic dwarfism, microcephaly

## Abstract

Cell number is a major determinant of organism size in mammals. In humans, gene mutations in cell cycle components result in restricted growth through reduced cell numbers. Here we identified biallelic mutations in *CDK4* as a cause of microcephaly and short stature. *CDK4* encodes a key cell cycle kinase that associates with D-type cyclins during G1 of the cell cycle to promote S-phase entry and cell proliferation through retinoblastoma (RB) phosphorylation. CDK4 and CDK6 are believed to be functionally redundant and are targeted jointly by chemotherapeutic CDK4/6 inhibitors. Using molecular and cell biology approaches, we show that functional CDK4 protein is not detectable in cells with *CDK4* mutations. Cells display impaired RB phosphorylation in G1, leading to G1/S-phase transition defects and reduced cell proliferation, consistent with complete loss of cellular CDK4 enzymatic activity. Together, these findings demonstrate that CDK4 is itself required for cell proliferation, human growth, and brain size determination during development.

## Abstract (precis)

In this study, Verdu Schlie et al. identify and characterize biallelic loss-of-function mutations in the cell cycle kinase CDK4 that cause microcephaly and growth deficiency in humans. The work elucidates CDK4's key role in G1-to-S transition (independent of mitosis) and cell proliferation during development.

Primary microcephaly (PM) is a Mendelian form of microcephaly where brain growth is markedly reduced during development and occurs in the absence of major malformations or neurological deficits (Verloes et al. 1993; Farcy et al. 2023). Mutations in multiple genes involved in centrosome and mitotic spindle biology were initially identified in primary microcephaly, leading to the proposal that defective neural stem cell mitosis causes microcephaly (Thornton and Woods 2009). However, other processes such as cell signaling, transcription, and the DNA damage response are also compromised in primary microcephaly (Faheem et al. 2015; Jayaraman et al. 2018), suggesting additional mechanisms of brain growth regulation.

Microcephalic dwarfism (MD) represents a group of genetic growth defects in which both brain and stature are compromised and is caused by mutations disrupting cell proliferation, genome stability, DNA replication, and mitosis (Klingseisen and Jackson 2011). Multiple primary microcephaly genes have subsequently been linked to microcephalic dwarfism phenotypes, indicating that these two conditions should be considered as a phenotypic continuum (Shaheen et al. 2019; Asif et al. 2023).

Alterations in cell cycle genes are major drivers of human cancer (Malumbres and Barbacid 2009). However, despite the availability of multiple mouse models, cyclins and CDKs have not been extensively studied in the context of human growth. Cyclin-dependent kinase 4 (CDK4) and CDK6 are functionally redundant regulators of G1 progression and the G1/S transition (Matsushime et al. 1994; Kato 1999; Sherr and Roberts 2004). CDK4 and CDK6 form complexes with D-type cyclins and link extracellular growth signals with cell cycle progression and growth (Malumbres and Barbacid 2009; Romero-Pozuelo et al. 2020; Fassl et al. 2022). CDK4/6 promotes phosphorylation and inactivation of the retinoblastoma (RB) tumor suppressor (Lundberg and Weinberg 1998; Harbour et al. 1999; Narasimha et al. 2014), resulting in E2F-driven transcription, increased CDK2/Cyclin E activity, and G1/S progression (DeGregori et al. 1995). Unsurprisingly given their antiproliferative properties, CDK4/6 inhibitors are used as therapies against multiple cancers (Fassl et al. 2022; Morrison et al. 2024).

Here we report the identification and functional characterization of biallelic loss-of-function (LOF) mutations in *CDK4* in individuals with microcephalic dwarfism. *CDK4* variants result in aberrantly spliced transcripts and undetectable full-length protein in patient fibroblasts, leading to impaired retinoblastoma phosphorylation in G1, delayed G1/S transition, and reduced proliferation to explain the growth deficiency observed in the affected individuals.

## Results

### Identification of biallelic variants in *CDK4*

**Figure 1.**
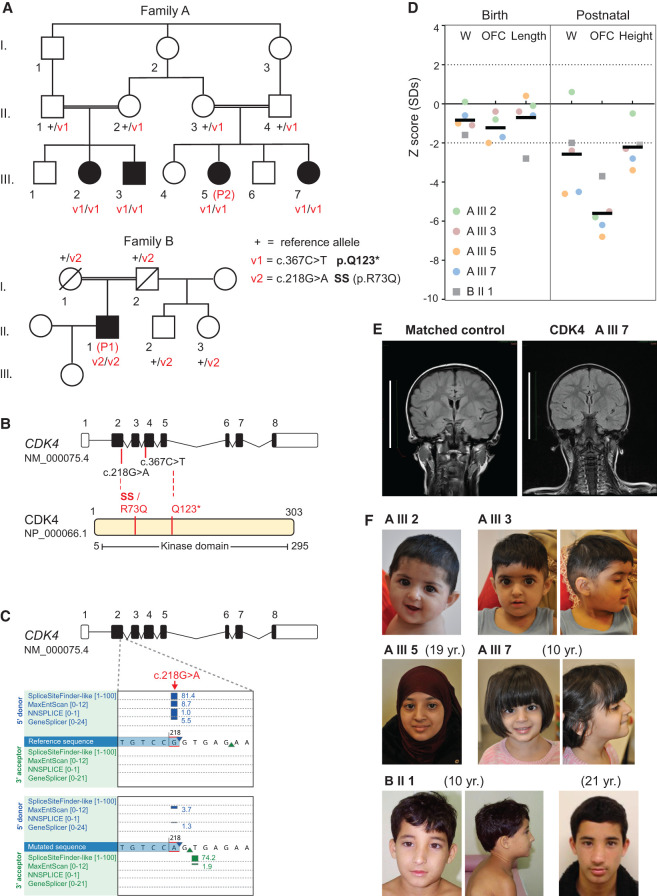
Individuals with biallelic *CDK4* variants display microcephaly and short stature. (*A*) Family pedigrees with segregation of *CDK4* variants. (Square) Male, (circle) female, (filled symbols) individuals with microcephaly, (strikethrough) deceased. WT Reference (+), variants v1 and v2, and zygosity are indicated for each studied individual. (*B*) Diagram of *CDK4* transcript (*top*) and protein (*bottom*); coding exons are depicted as black rectangles. Red lines indicate variant location. (SS) Splice site disrupted. (*C*) Altered splicing predictions for the c.218G > A substitution generated using Alamut. (Blue rectangles) Strength of splice donor predictions for individual splice algorithms, (blue triangle) predicted donor splice site. (*D*) Growth parameters at birth and at last assessment (postnatal). (W) Weight, (OFC) orbito–frontal circumference. Z-scores show standard deviations from population mean for age and sex. Dashed lines indicate a 95% confidence interval for the general population. Individual subject data points from families A (circles) and B (squares) are graphed, and mean values are plotted. (*E*) MRI scan of age-matched control (4 years 8 months) and affected individuals with a *CDK4* variant. Coronal FLAIR projection shows simplified parietal and temporal gyri, reduced white matter volume, and the absence of brain malformations. Scale bars, 10 cm. (See also Supplemental Figure S1C for additional MRI projections.) (*F*) Photographs of all affected individuals.

We ascertained a consanguineous family of British Pakistani origin with four affected individuals presenting with extreme microcephaly and (for three out of four) significantly short stature (Fig. 1A; Tables 1, 2). Whole-genome sequencing of parents and affected individuals revealed the homozygous substitution c.367C > T in cyclin-dependent kinase 4 (*CDK4*;NM_000075.4) as the likely cause of the phenotype. This variant introduces a premature stop codon (p.Q123*) within the kinase domain of the protein (Fig. 1B), with its position in exon 4 of eight predicting it also to result in nonsense-mediated decay of the transcript.

**Table 1.** *Biallelic* CDK4 *variants in individuals with microcephaly and short stature*

Family	Individual	Sex	Nucleotide change	Amino acid consequence	Allele frequency (gnomAD)	Segregation	Consanguinity	OFC (SD)	Height (SD)	Intellectual disability#	Age at exam	Other
A	A.III.2	Female	c.367C > T	p.Gln123*	6.20 × 10^−07^	M, P	Yes	−5.8	−0.5	Mild	11 years 8 months	Autoimmune hypothyroidism
A	A.III.3	Male	c.367C > T	p.Gln123*	6.20 × 10^−07^	M, P	Yes	−5.5	−2.3	Mild	6 years 1 month	Autoimmune neutropenia
A	A.III.5 (P2)	Female	c.367C > T	p.Gln123*	6.20 × 10^−07^	M, P	Yes	−6.8	−3.4	Mild	19 years 6 months	
A	A.III.7	Female	c.367C > T	p.Gln123*	6.20 × 10^−07^	M, P	Yes	−6.2	−2.8	Mild	10 years 3 months	Celiac disease, autoimmune hypothyroidism, pons hypoplasia
B	B.II.1 (P1)	Male	c.218G > A	p.R73Q and loss of essential donor splice site	1.86 × 10^−06^	M, P	Yes	−3.7	−2.1	No	33 years 8 months	Epilepsy, anemia, low reticulocytes

Variants are described using Human Genome Variation Society (HGVS) nomenclature (https://hgvs-nomenclature.org/stable/recommendations/general) for the reference coding DNA (HGVSc; NCBI reference sequence NM_00075.4) and protein (HGVSp; NCBI reference sequence NP_000066.1) sequences. Variant allele frequency for the total gnomAD v4.1 population. DNA variants are expressed relative to the coding (c.) sequence, and all protein changes are preceded by “p.” OFC and height at latest evaluation are shown. (#) Additional clinical information in Table 2. (M) Maternal, (P) paternal.

**Table 2.** *Detailed clinical features of individuals with* CDK4 *variants and microcephaly*

Individual	A.III.2	A.III.3	A.III.5	A.III.7	B.II.1
*CDK4* variant	c.367C > T; p.Gln123Ter	c.367C > T; p.Gln123Ter	c.367C > T; p.Gln123Ter	c.367C > T; p.Gln123Ter	c.218G > A
Ethnicity	British Pakistani	British Pakistani	British Pakistani	British Pakistani	Brazilian
Consanguinity	Yes (first)	Yes (first)	Yes (first)	Yes (first)	Yes (first)
Mid-parental height SDS	160 cm/−0.6 SD	173 cm/−0.6 SD	155 cm/−1.4 SD	155 cm/−1.4 SD	180 cm/0.8 SD
Sex	Female	Male	Female	Female	Male
Current age	11 years	6 years	19 years	10 years	33 years
Prenatal-onset growth restriction	No; maternal gestational diabetes	No	No	No	Yes
Gestational age	40 weeks	40 weeks	38 weeks	40 weeks	39 weeks
Birth weight SDS	3.44 kg/0.1 SD	3.08 kg/−1.1 SD	2.6 kg/−1.0 SD	3.18 kg/−0.6 SD	2.68 kg/−1.6 SD
Birth length SDS	53.5 cm (at 1 month)/−0.1 SD	50 cm/−0.4 SD	49.5 cm/0.4 SD	49 cm/−0.6 SD	44 cm/−2.8 SD
Birth OFC SDS	33.5 cm/−0.8 SD	34 cm/−0.4 SD	32 cm/−2.0 SD	32.5 cm/−1.7 SD	NA
**At the first evaluation**					
Postnatal growth retardation	Yes	Yes	Yes	Yes	Yes
Microcephaly	Yes	Yes	Yes	Yes	Yes
Chronological age	1 years	0.75 years	0.83 years	4.58 years	4.1 years
Height SDS	67 cm/−2.8 SD	62 cm/−4.1 SD	66 cm/−2.2 SD	92.4 cm/−3.1 SD	92.5 cm/−2.5 SD
Weight or BMI SDS	7.3 kg/−1.7 SD	6.98 kg/−2.2 SD	5.66 kg/−3.4 SD	11.8 kg/−3.4 SD	13.3 kg/−2.1 SD
OFC SDS	39.9 cm/−5.6 SD	40.4 cm/−5.1 SD	37.5 cm/−7.1 SD	43.9 cm/−6.3 SD	43.5 cm/−6.0 SD
Age at most recent exam	11 years 8 months	6 years 1 month	19 years 6 months	10 years 3 months	32 years 8 months
Recent height	144.4 cm/−0.5 SD	105 cm/−2.3 SD	143.4 cm/−3.4 SD	121.5 cm/−2.8 SD	163.7 cm/−2.1 SD^a^
Recent weight	43.6 kg/0.6 SD	15.85 kg/−2.4 SD	33.39 kg/−4.6 SD	17.4 kg/−4.5 SD	54.6 kg/−2.0 SD
Recent OFC	46.8 cm/−5.8 SD	44.8 cm/−5.5 SD	46.1 cm/−6.8 SD	46.0 cm/−6.2 SD	51.0 cm/−3.7 SD
Developmental delay	Learning disability diagnosed on formal cognitive assessment^b^	Speech delay, behind peers at school	Mild to moderate learning disability diagnosed on formal cognitive assessment	Learning disability diagnosed on formal cognitive assessment	No
Dysmorphic features/congenital malformations	Low insertion of columella, three café au lait patches	Low insertion of columella, two café au lait patches	Preauricular skin tag	—	Shawl scrotum, small testicles in adulthood
Other clinical features	Autoimmune hypothyroidism	Consumptive neutropenia	Growth did not respond to growth hormone, delayed bone age (4.0) at chronological age 8.0	Celiac disease, delayed bone age (2.8) at chronological age 4.5, and bone age 5.0 at chronological age 7.5; autoimmune hypothyroidism	Epilepsy during childhood; normocytic anemia with low reticulocyte^c^
**Laboratory findings**					
FT4 (pmol/L)	**8.2** (*n* range 12–22)	15 (*n* range 10–18)	10 (*n* range 10–19)	11.4 (*n* range 12–22)	14–20 (*n* range 10–19)
TSH (mU/L)	**5.1** (*n* range 0.27–4.21)	3.4 (*n* range 0.5–4.2)	3.41 (*n* range 0.5–3.9)	8.7 (*n* range 0.27–4.2)	7.7–12.4 (*n* range 0.4–4.5)
GH peak at a stimulation test (µg/L)	NA	NA	20	NA	8.8
IGF-1 (ng/L)	NA	93 (*n* range 28–247)	115 (*n* range 35–240)	51 (*n* range 25–198)	**46** (*n* range 81–280)
LH/FSH (IU/L)	5.7/5.5	NA	NA	NA	35.7/18.4^d^
Other laboratories	Antithyroid peroxidase 204.0 IU/mL (*n* range < 34.0); urine OA, AA, MPS normal; Fanconi breakage studies normal	Granulocyte-specific antibodies; normocellular marrow with adequate myeloid precursors that are maturing to segmented neutrophils	HbA1c 37 mmol/mol (*n* range 20–41); glucose 4.3 mmol/L (*n* range 3.3–6.1)	Antithyroid peroxidase 165.0 IU/mL (*n* range < 34.0); HbA1c 37 mmol/mol (*n* range 20–41); random glucose 5.1 mmol/L (*n* range 3.3–6.1)	Normal blood glucose levels with an HbA1c of **5.7%–6.0%**
Brain MRI findings	NA	NA	Brain MRI normal	Microcephaly with simplified gyri. Hypoplastic pons with relatively normal cerebellum. Dorsal and ventral midline clefts of pons and medulla, small cerebellar peduncles	Rathke cleft cyst resolved^e^

(NA) data not available.

^a^Adult height after 5.6 years of rhGH therapy.

^b^A.III.2: Psychology assessment meets criteria for learning disability.

^c^Normal hemoglobin electrophoresis and iron status.

^d^In puberty and adulthood with normal testosterone levels (18.4–28.8 nmol/L [normal range 10–35 nmol/L]) and spermogram count and normal fertility.

^e^Rathke cleft cyst was observed at the age of 8 and completely disappeared during the follow-up (last image at the age of 18).

Through GeneMatcher (Sobreira et al. 2015), we then identified an individual of Brazilian origin with a history of prenatal-onset growth restriction who also presented with marked microcephaly and short stature (Fig. 1A,B; Tables 1, 2). Independent whole-exome sequencing identified a homozygous c.218G > A variant in *CDK4*. This results in an arginine-to-glutamine substitution at codon 73 (p.R73Q), a conserved residue in mammals and most vertebrates (Supplemental Fig. S1A). Significantly, this nucleotide substitution also occurs at an essential splice site sequence: the last base pair of exon 2. Analysis of this variant using SpliceAI (Jaganathan et al. 2019), MaxEntScan, and NNSPLICE predicts loss of the splice donor site located 1 bp downstream from the substitution (Fig. 1B,C). Consequently, loss of exon 2, resulting in the loss of the canonical start site, would be anticipated to significantly disrupt protein function.

The presence of homozygous *CDK4* variants was confirmed by Sanger sequencing of blood genomic DNA in all affected individuals. Parents were heterozygous carriers (Fig. 1A; Supplemental Fig. S1B); hence, variant segregation in the families was in keeping with autosomal recessive inheritance. Consistent with a rare autosomal recessive disorder, both variants were observed at very low frequency in gnomAD (v4.1.0). Allele frequencies were 6.2 × 10^−7^ and 1.9 × 10^−6^, respectively, and only observed in the heterozygous state. Constraint data from gnomAD indicate that *CDK4* is intolerant to biallelic loss-of-function variants (pNull 5 × 10^−4^), supporting the pathogenicity of these variants.

Clinically, all individuals presented with extreme microcephaly (occipital–frontal circumference [OFC] –5.6 ± 1 SD), and most had significant postnatal growth restriction (height –2.22 ± 0.97 SD) (Fig. 1D; Tables 1, 2). At birth, growth parameters were mildly reduced (OFC –1.23 ± 0.65 SD; birth length –0.7 ± 1.1 SD) but were within normal population limits. Neuroimaging studies demonstrated a significant reduction of brain size and simplified cortical gyration evident on coronal and axial projections (Fig. 1E; Supplemental Fig. S1C), in keeping with the microcephaly with simplified gyri seen in primary microcephaly (Verloes et al. 1993; Passemard et al. 2009). Cortical structural abnormalities were not evident. A hypoplastic pons without reduction in cerebellar size was reported in A.III.7 (Supplemental Fig. S1C). However, this was a variable feature, as microcephaly with simplified gyri and no brainstem alteration was reported in individual A.III.5.

Mild to moderate intellectual disability was manifest in the four affected individuals from family A, whereas individual B.II.1, with the mildest microcephaly (−3.7 SD), had normal cognitive function. No distinctive dysmorphism or malformations were evident across the two families. Siblings A.III.2 and A.III.3 had three and two cafe au lait patches, respectively. They also had low insertion of the columella but were facially otherwise unremarkable (Fig. 1F). A.III.2 and an unaffected sibling had autoimmune hypothyroidism. A.III.3 had autoimmune-mediated neutropenia. Siblings A.III.5 and A.III.7 both presented delayed bone age, whereas A.III.7 had celiac disease and autoimmune hypothyroidism (Tables 1, 2). Individual B.II.1 developed epilepsy during childhood and exhibited mild microcytic/normocytic anemia of undefined etiology that resolved spontaneously after puberty (Supplemental Table S1). During a long follow-up period, slightly elevated TSH values with normal thyroid hormone and ultrasound and negative antibodies were observed (Table 2). He had a shawl scrotum, normal-sized penis, and normally positioned urethra. The patient entered puberty at the age of 11, with elevated gonadotropin and normal testosterone levels. In adulthood, he has reduced testicular volume (11 mL) and normal sperm analysis and has recently fathered a healthy child.

Altogether, we identified homozygous variants in two phenotypically similar families, with affected individuals displaying nonsyndromic microcephaly and short stature, which appeared likely to result in abrogation of CDK4 function. We therefore proceeded to determine the functional impact of the *CDK4* variants at the cellular level.

### Transcriptional consequences of *CDK4* mutations

**Figure 2.**
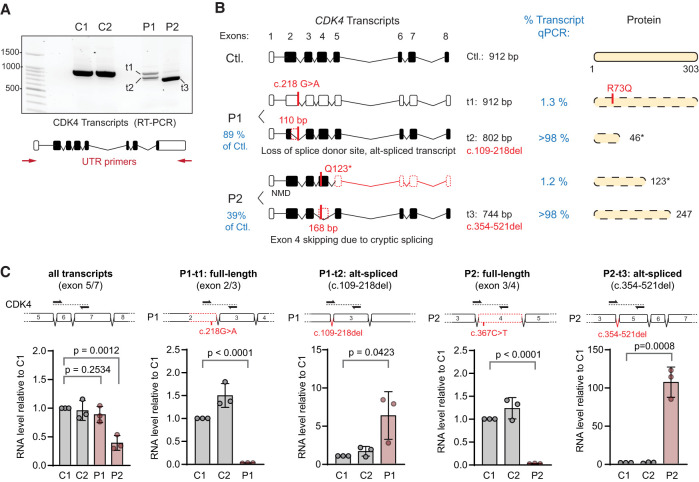
Transcriptional consequences of *CDK4* mutations. (*A*) Transcript analysis by RT-PCR of RNA extracted from primary fibroblasts. Agarose gel electrophoresis of RT-PCR products using *CDK4* 5′ and 3′ UTR primers. A full-length (t1) transcript of 912 bp and a shortened (t2) one were seen in P1 (v2), whereas P2 (v1) exhibited a predominant smaller transcript (t3). (*B*) Schematics of detected transcripts and their relative quantification (percent transcript) based on qPCR results presented in *C*; the corresponding predicted proteins are shown at the *right*. Supplemental Figure S2 presents Sanger sequences of cloned *CDK4* transcripts after RT-PCR. (*C*) qPCR analysis of WT control (C1 and C2) and patient-specific *CDK4* transcripts relative to control. Primer locations for each qPCR reaction are indicated *above* each bar graph. *n* = 3 experiments; mean ± SEM; two-tailed *t*-tests.

To assess the consequence of these candidate variants on *CDK4* transcriptional levels and splicing, we established primary fibroblast cell lines from affected individuals P1 (B.II.1) and P2 (A.III.5). We investigated *CDK4* transcript levels and splicing by reverse transcription and PCR amplification (RT-PCR) of RNA extracted from P1 and P2 fibroblasts using 5′ and 3′ UTR primers. This demonstrated the presence of shorter *CDK4* transcripts that were not present in wild-type controls (Fig. 2A). Subsequent cloning and Sanger sequencing of these PCR products demonstrated two transcripts (t1 and t2) in P1, one full-length *CDK4* (912 bp) containing the c.218 G > A point substitution, and a smaller 802 bp fragment with a 110 bp deletion comprising bases 109–218, corresponding to most of exon 2 (Fig. 2A,B; Supplemental Fig. S2A,B). The donor splice site located 1 bp downstream from c.218 was disrupted, as predicted by in silico analysis (SpliceAI, Δ = 0.79) (Fig. 1C). The resulting open reading frame led to a frameshift encoding a truncated length polypeptide (46 amino acids; r.109_218del; p.Val36Alafs10) missing almost the full kinase domain (Fig. 2B; Supplemental Material). Further qPCR analysis supported the truncated polypeptide being the major transcript in this patient (Fig. 2C). In contrast, full-length transcripts containing the c.218 G > A substitution (encoding the p.R73Q change) were present at negligible levels (1.3% of wild type) (Fig. 2C, second panel). Nevertheless, if it were to contribute to CDK4 protein production, the R73 side chain participation in salt bridges would be disrupted by this mutation, suggesting that any protein derived from this R73Q allele would be functionally compromised (Supplemental Fig. S1D).

For P2, the c.367C > T substitution encoding stop codon p.Q123* was expected to cause nonsense-mediated decay, and consistent with this, a full-length transcript (912 bp) was nearly undetectable by RT-PCR (Fig. 2A). Surprisingly, however, a shorter transcript was present at ∼31% of wild-type transcript levels when quantified by qPCR (Fig. 2C, first graph). Sequencing of this 744 bp PCR product demonstrated a 168 bp deletion from c.354 to c.521, corresponding to exon 4 (Fig. 2B; Supplemental Fig. S2C). Thus, the predominant *CDK4* transcript in P2 cells results in an in-frame deletion with loss of 56 amino acids (p. Asp119_Val174del) (Fig. 2C, last two panels). Mapping of these 56 amino acids on the CDK4 structure shows that a key region of CDK4, including the essential activation segment (containing Thr172, which is critical for activity), is missing, likely leading also to a loss of protein stability (Supplemental Fig. S1E).

### CDK4 kinase is undetectable in patient-derived cells

**Figure 3.**
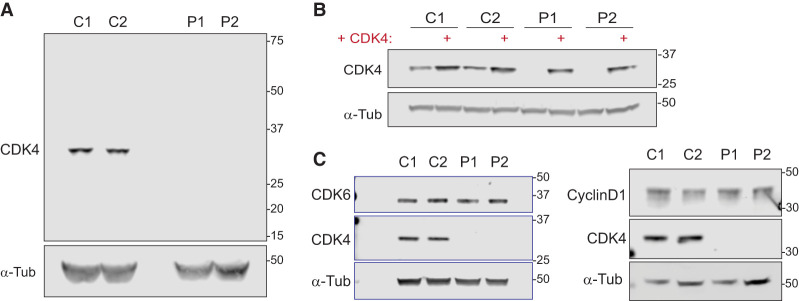
Full-length CDK4 protein is undetectable in patient fibroblasts. (*A*,*B*) Immunoblots of total cell extracts obtained from exponentially growing control (C1 and C2) and patient (P1 and P2) fibroblasts without (*A*) and with (*B*) CDK4 complementation. α-Tubulin was used as the loading control. A rabbit monoclonal antibody to C-terminal CDK4 was used; a different mouse CDK4 antibody raised against full-length CDK4 was used in Figure 5A. A smaller ∼12 kDa molecular weight band was variably detected in P1 with this antibody (Supplemental Fig. S2D) that might correspond to the 46 amino acid truncated nonfunctional protein predicted from RNA studies. (*C*) CDK6 and Cyclin D1 levels were unchanged in patient fibroblasts compared with wild-type controls.

We next assessed the expected consequences on cellular protein levels experimentally. Here, Western blot analysis of total cell extracts showed undetectable levels of full-length CDK4 protein in patient-derived cells from both families in contrast to control fibroblasts (Fig. 3A). CDK4 complementation confirmed specificity of CDK4 antibodies (Fig. 3B). Using another antibody raised against full-length CDK4, a smaller protein migrating between 8 and 15 kDa was detected in P1 fibroblasts on some blots but not others. This might conceivably correspond to the 46 amino acid polypeptide (expected molecular weight 5 kDa) predicted for P1-t2, (Supplemental Fig. S2D).

In conclusion, for P1, RNA and protein analysis confirmed near-complete disruption of the exon 2 splice donor site and demonstrated that the major remaining transcript had a much shorter open reading frame that led to early truncation of the CDK4 protein. Therefore, this mutation resulted in no detectable full-length CDK4 protein by immunoblotting, though expression of a small truncated CDK4 protein fragment without expected functionality cannot be completely excluded. For P2, the full-length transcript containing the premature stop codon was markedly reduced, and the alternate transcript caused an in-frame deletion. Neither transcript produced an active kinase, and therefore, for P2, this mutation, like for P1, should be functionally null—at the very least in terms of its canonical activity as a kinase.

### Normal mitosis in CDK4 mutant cells

A homozygous *CDK6* missense mutation (p.Ala197Thr) was previously reported in a family with primary microcephaly. Although this variant did not affect CDK6 stability, centrosomal localization of CDK6 observed in control fibroblasts was reported as lost in *CDK6* p.Ala197Thr patient cells. Additionally, patient cells displayed disorganized mitotic spindles and microtubules, supernumerary centrosomes, and nuclei with abnormal morphology, leading the investigators to propose that centrosome and microtubule dysfunction contributed to the proliferation defect of *CDK6* mutant cells (Hussain et al. 2013).

**Figure 4.**
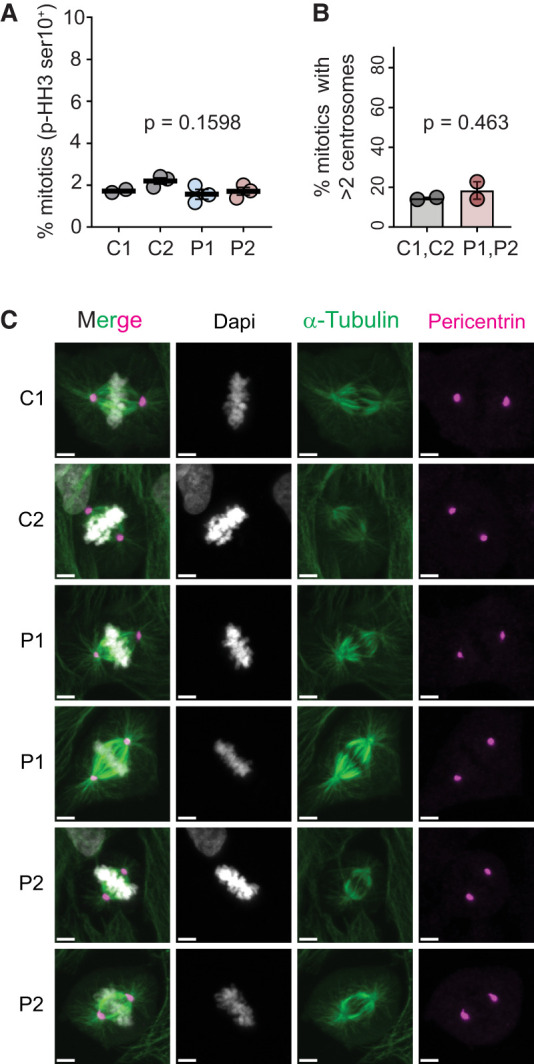
*CDK4* mutations do not alter mitosis. (*A*) Percentage of mitotic cells (p-Histone H3 ser10-positive) in control (C1 and C2) and patient (P1 and P2) fibroblasts as measured by flow cytometry. Data points are from three independent experiments (two for C1); one-way ANOVA with Tukey post test; mean ± SEM. (*B*) Quantification of metaphase cells with more than two centrosomes, expressed as percentage. Numbers of cells analyzed were as follows: C1, 79; C2, 94; P1, 150; and P2, 101. Two-tailed *t*-test; mean ± SEM; measurements were pooled from two independent experiments. (*C*) Representative confocal images of control (C1 and C2) and patient (P1 and P2) fibroblasts fixed and stained for DAPI (gray), α-tubulin (green), and pericentrin (magenta). Scale bars, 5 µm.

Given the functional overlap of CDK6 and CDK4, we investigated the possibility of centrosome/mitotic defects in *CDK4* mutant cells. However, the proportion of mitotic cells (p-Histone H3-positive) was similar between control and patient-derived fibroblasts, suggesting that mitosis progression is unaffected (Fig. 4A). Additionally, in contrast to CDK6, CDK4 did not associate preferentially with centrosomes in human primary fibroblasts (Supplemental Fig. S3). Furthermore, mitotic spindles appeared normal, as assessed by α-tubulin and pericentrin immunofluorescence, and we failed to detect supernumerary centrosomes in patient cells (Fig. 4B,C). Therefore, mitotic defects were unlikely to be the cause of the growth defect caused by *CDK4* microcephaly mutations.

### *CDK4* mutations impair cell proliferation and the G1/S transition

**Figure 5.**
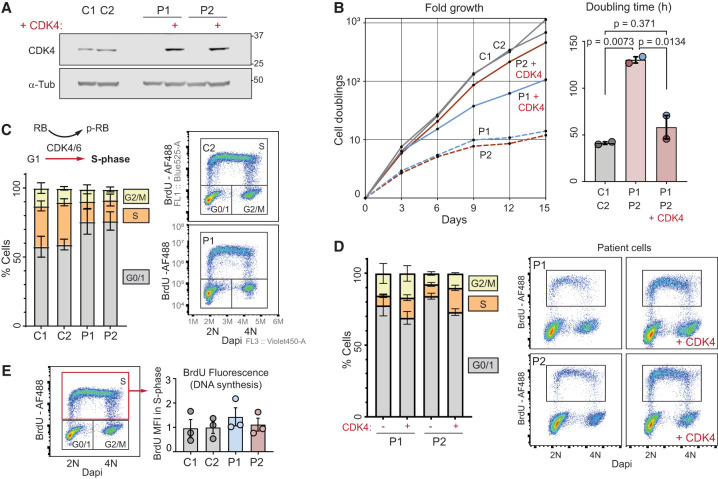
*CDK4* mutations impair G1-to-S progression and lead to reduced cell proliferation. (*A*) Western blot of control and patient-derived fibroblasts with and without WT CDK4 complementation. (*B*, *left*) Growth curves of control and patient-derived fibroblasts with and without WT CDK4 complementation. (*Right*) Bar graph showing quantification of doubling times; one-way ANOVA with Tukey post test. *P*-values are indicated; mean ± SEM. (*C*) Cell cycle distribution (G0/G1, S, and G2/M) derived from BrdU and DNA (DAPI) flow cytometry scatter plots show fewer cells in S phase (BrdU^+^) in patient-derived fibroblasts compared with controls. *n* = 3 independent experiments; mean ± SEM. Gates are shown on representative plots at the *right*. (*D*) Cell cycle distribution after complementation of patient-derived fibroblasts with CDK4. Reduced G0/G1 and increased S-phase populations consistent with rescue of a G1/S progression defect. *n* = 3 independent experiments; mean ± SEM. (See also Supplemental Fig. S4A.) (*E*) Quantification of DNA synthesis rate (BrdU mean fluorescence intensity [MFI] of gated population in the red rectangle) from experiments depicted in *C*.

Given CDK4's canonical role in cell cycle progression (Baker et al. 2022; Fassl et al. 2022), we next investigated whether the *CDK4* mutations lead to reduced cell proliferation. Growth rates were determined for patient-derived fibroblasts with and without CDK4 complementation (Fig. 5A,B). CDK4-deficient cells proliferated three times slower than control fibroblast cell lines (doubling times: C1 = 43 h, C2 = 40 h, P1 = 127 h, and P2 = 133 h). Importantly, complementation of patient cells significantly rescued proliferation, establishing CDK4 deficiency as the cause of the cell proliferation defect (Fig. 5A,B).

Because the outcome of CDK4/6 kinase function is G1-to-S-phase transition (Chung et al. 2019; Yang et al. 2020), *CDK4* mutations would most likely result in the accumulation of cells in G1 (Tsutsui et al. 1999), consistent with a longer cell cycle in CDK4-deficient cells. Indeed, flow cytometry experiments using a 40 min BrdU pulse to label replicating cells demonstrated that CDK4-deficient fibroblasts accumulate in G0/G1 and have proportionately fewer S-phase cells compared with control lines (Fig. 5C). Moreover, CDK4 complementation attenuated this G1 accumulation and enhanced S-phase cell numbers (Fig. 5D; Supplemental Fig. S4A). S phase itself did not appear to be impacted, with normal levels of DNA synthesis measured by BrdU incorporation with equal mean fluorescence intensity (MFI) in S phase for wild-type and CDK4-deficient cells (Fig. 5E). Moreover, serial labeling of cells with BrdU and EdU did not detect a difference in S-phase length (Supplemental Fig. S4B). Therefore, loss of CDK4 leads to an extended G1 phase in patient cells, without detectable impact on S phase and DNA replication. CDK6 also promotes G1/S transition, with its overexpression compensating for CDK4 loss (Supplemental Fig. S5A–C). However, CDK6 and Cyclin D1 levels were unchanged in CDK4 mutant cells (Fig. 3C), and therefore such compensation did not occur in patient cells. Nevertheless, CDK6 activity within these cells was necessary for CDK4-deficient cells to progress from G1 to S, as treatment with the CDK4/6 inhibitor palbociclib or CDK6 depletion by RNAi prevented DNA replication, as measured by EdU incorporation (Supplemental Fig. S5D–F).

**Figure 6.**
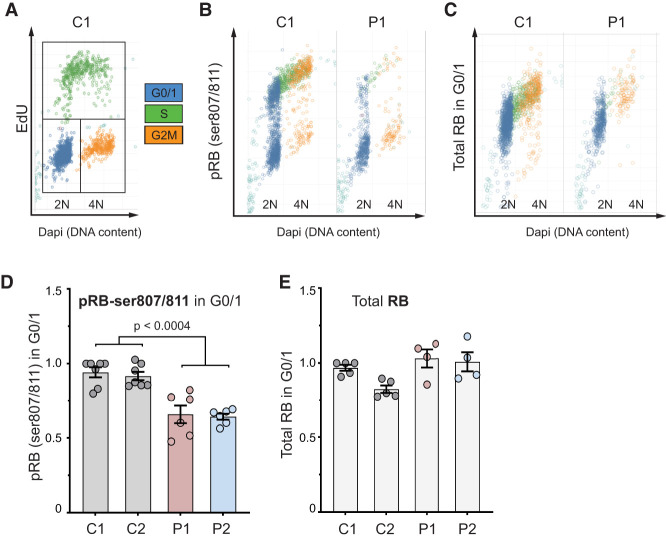
*CDK4* mutations impair retinoblastoma phosphorylation in G1. (*A*–*E*) Quantitative image-based cytometry (QIBC). (*A*) Gating strategy for cell cycle stages by DNA content (DAPI) and EdU incorporation. (*B*) Representative DAPI versus pRB-ser807/811 scatter plots demonstrate impaired RB phosphorylation in G1 in CDK4-deficient cells (P1) relative to control 1. (*C*) Representative scatter plot of total RB levels demonstrating equivalent levels of RB between C1 and P1. Data points for *C* and *D* individual cells: *n* > 1500 cells/sample in each independent experiment. (*D*,*E*) Quantification of pRB-ser807/811 (*D*) and total RB (*E*) fluorescence intensity per nucleus show significantly reduced pRB-ser807/811 and normal total RB levels in G0/G1 in CDK4-deficient fibroblasts relative to controls. Mean ± SEM; *n* ≥ 4 independent experiments with 72 images/condition, totaling ≥1500 cells/sample or condition in each experiment analyzed.

G1 transition to S phase is regulated by retinoblastoma (RB) phosphorylation by CDKs (Harbour et al. 1999; Narasimha et al. 2014), which leads to derepression of E2F transcription factors and expression of S-phase proteins such as Cyclin E and CDC25A (Leone et al. 1998; Bracken et al. 2004). Consistent with CDK4 loss impacting E2F-dependent transcription, qRT-PCR demonstrated reduced *CDC6* and *PCNA* expression (Supplemental Fig. S4C). Likewise, complementation of CDK4-deficient fibroblasts with wild-type CDK4 significantly increased the expression of these E2F target genes (Supplemental Fig. S4D). We next assessed RB phosphorylation, where, to ensure that the cell cycle was not a confounder, quantitative image-based cytometry (QIBC) (Toledo et al. 2013) was performed using a validated immunofluorescence methodology to assess pRB and RB levels in individual cells (Chung et al. 2019). Using this approach, analysis of G0/G1-phase cells demonstrated a significant reduction of pRB but not RB in CDK4-deficient cells before S-phase onset, when RB phosphorylation is CDK4/6-dependent (Fig. 6A–E). pRB levels in G0/G1 were rescued by CDK4 complementation and were sensitive to CDK4/6 inhibition (Supplemental Fig. S6A–D), supporting this conclusion. In S phase, pRB levels in individual cells were similar to wild-type cells (Supplemental Fig. S6E), likely due to the subsequent feed-forward activation of CDK2–CyclinA/E augmenting RB phosphorylation (Knudsen and Knudsen 2008). This could account for normal levels of DNA synthesis and S-phase duration in CDK4-deficient cells (Fig. 5E; Supplemental Fig. S4B). Together, these results indicate that the canonical function of CDK4 regulating the G1-to-S transition through phosphorylation of RB in G1 underlies the cell proliferation defect caused by *CDK4* mutations.

## Discussion

CDK4 and CDK6 are functionally redundant kinases promoting cell cycle progression and the G1/S transition, linking extracellular growth signals with cell proliferation (Malumbres and Barbacid 2009; Fassl et al. 2022). Many cancer types are dependent on these activities for growth (Gao et al. 2020), and small molecule CDK4/6 inhibitors are widely studied in clinical trials or have shown success in the treatment of some cancer types (Fassl et al. 2022; Morrison et al. 2024).

Previously, heterozygous germline mutations in *CDK4* (R24C or R24H) causing gain of function were described in familial melanoma cases (Zuo et al. 1996; Puntervoll et al. 2013). These reduce CDK4 binding to p16^INK4^, leading to constitutive CDK4 kinase activity (Wölfel et al. 1995). Here we present evidence for CDK4 as a regulator of human brain and organism growth, identifying homozygous loss-of-function *CDK4* mutations in individuals from two independent families with postnatal growth restriction and severe microcephaly. *CDK4* mutations resulted in no detectable functional protein in patient fibroblasts, impairing RB phosphorylation and G1/S transition. These findings indicate that these mutations are loss of function, and that canonical CDK4 kinase dysfunction is likely responsible for the cell proliferation defect causing growth deficiency in affected individuals. Therefore, human CDK4 is not redundant with CDK6 for growth during development.

The extreme microcephaly in *CDK4* individuals as well as the mild intellectual disability are characteristic features of primary microcephaly (Farcy et al. 2023). However, growth parameters at birth, including OFC, were only 1–2 SD below population mean. Notably, heterozygous frameshift and nonsense variants in *CCND2* (encoding Cyclin D2), the partner of CDK4/6 with major roles in neural progenitor proliferation (Glickstein et al. 2009), also result in microcephaly (Pirozzi et al. 2021). *CDK4* and most *CCND2* microcephaly individuals display similar growth restriction, with reduced growth parameters at birth of −1 to −2 SD but more significant microcephaly postnatally (Pirozzi et al. 2021), suggestive of a prenatal-onset origin with a delayed presentation. This trajectory of early postnatal brain growth restriction has also been reported in individuals affected by paradigmatic primary microcephaly genes such as *ASPM* (Passemard et al. 2009). Therefore, although strictly not fulfilling the criteria for “primary,” where microcephaly is evident at birth, the *CDK4* phenotype otherwise parallels primary microcephaly and overlaps with microcephalic dwarfism.

*Cdk4* knockout mice display significantly reduced body size, impaired fertility, and insulin-deficient diabetes due to abnormal postnatal islet cell development (Rane et al. 1999; Tsutsui et al. 1999; Moons et al. 2002; Martin et al. 2003; Mettus and Rane 2003). However, B.II.1 was fertile, and although he had borderline glycosylated hemoglobin levels, none of the individuals assessed had a diagnosis of diabetes mellitus. In family A, several members had autoimmune disorders (hypothyroidism or neutropenia). However, these conditions did not entirely cosegregate with affected status and were not reported in family B. So, they are most likely coincidental, reflecting familial aggregation for an independently acting polygenic predisposition. Cell size was not reduced in organs from *Cdk4* KO mice, indicating a primary cell proliferation defect as the source of dwarfism (Martin et al. 2003). In contrast, *Cdk4*-R24C gain-of-function (GOF) mice display increased proliferation, body size, and cancer risk (Rane et al. 2002), supporting the notion that CDK4 is a bona fide regulator of cell number in mammals. Although present at birth, the growth defect in *Cdk4* KO mice becomes more severe during postnatal development (Rane et al. 1999; Tsutsui et al. 1999). Hence, progressive growth restriction during development is a consistent phenotype between mice and humans.

With CDK6 primary microcephaly previously reported (Hussain et al. 2013), discovering CDK4 mutations as a cause for microcephaly seems on the face of it unsurprising. However, despite substantial overlap in function during G1-phase progression, there are significant differences in disease mechanisms for growth and microcephaly associated with mutations in these two kinases. Here, we demonstrate that human *CDK4* primary cells have absent functional protein, impaired G1/S progression, and cell proliferation not compensated for by endogenous CDK6 activity. Hence, resulting hypocellularity likely accounts for microcephaly and growth failure in *CDK4* cases (Klingseisen and Jackson 2011). Notably, such short stature does not occur in *CDK6* primary microcephaly (Hussain et al. 2013). Likewise, in mice, rather than a 50% reduction in *Cdk4* adult size (Rane et al. 1999), body weight is only slightly reduced in *Cdk6* females (Malumbres et al. 2004). Also, the homozygous Ala197Thr missense mutation does not affect CDK6 protein stability, and an alternative pathogenic role for CDK6 in centrosome and microtubule organization was proposed as causal for microcephaly (Hussain et al. 2013). Although Cdk6 localization to centrosomes was not detected in radial glia, the Ala197Thr mutation was found to impair outer radial glia (oRG) progenitor expansion (Wang et al. 2022). Such expansion was driven by Cdk6 in a noncatalytic manner and was not impacted by Cdk4 deletion (Wang et al. 2022). Therefore, extreme microcephaly observed in both *CDK6* and *CDK4* cases do not share a common mechanism.

Experimental manipulation of G1 length through Cdk4/CyclinD1 overexpression or shRNA depletion alters the balance of progenitor self-renewal and neurogenesis, altering surface area of the postnatal cerebral cortex (Lange et al. 2009); thus, a role for CDK4 in regulating G1 progression in neurogenesis would be sufficient to explain microcephaly. However, because these experiments included the simultaneous overexpression/depletion of Cyclin D, they do not preclude a role for the CDK6 kinase.

Despite the functional equivalence of Cyclin D1, D2, and D3 in supporting CDK activity, D-type Cyclin knockouts have differing developmental consequences (Ciemerych et al. 2002; Glickstein et al. 2009; Saleban et al. 2023). This may be explained at least in part by different tissue expression patterns. Likewise, interrogation of a published scRNA-seq data set (Telley et al. 2019) demonstrates distinct expression patterns for CDK4 and CDK6 during neurogenesis (Supplemental Fig. S7). Notably, CDK6 expression is substantially more restricted to early progenitors, with CDK4 expression persisting longer during neurogenic differentiation. This raises the possibility that loss of CDK4 may become limiting later in neurogenesis when CDK6 expression is minimal or absent. Also, the predominantly postnatal nature of *CDK4* microcephaly suggests that other processes contributing to brain volume could be impacted, such as gliogenesis, which peaks in the third trimester and continues postnatally (Spalding et al. 2005; Gilmore and Walsh 2013). Furthermore, CDK4 might have a noncanonical role analogous to the role of CDK6 in cilia regulation (He et al. 2025) or the unconventional role of cyclin-dependent kinase CDK5 in postmitotic neurons (Tsai et al. 1993).

With LOF mutations in the CDK4/6 binding partner *CCND2* causing microcephaly (Pirozzi et al. 2021), and GOF *CCND2* mutations leading to brain overgrowth (Mirzaa et al. 2014), we conclude that CDK4 and G1 Cyclin–CDK activity represents a key axis controlling human brain growth. A limitation of our study is that CDK4 loss has not been investigated in neural progenitors; hence, its neurodevelopmental mechanism remains to be confirmed, and the cell populations impacted have yet to be defined. Cortical size is much more subtly affected in microcephaly mouse models (Pulvers et al. 2010) and has not been investigated in *Cdk4* KO mice. Given our findings in humans here, re-evaluation of in vivo neurogenesis in *Cdk4*^−/−^ mice is warranted, as a role for CDK4 in corticogenesis may have been previously overlooked.

## Materials and methods

### Research subjects

Patients were recruited to research studies at the MRC Human Genetics Unit, University of Edinburgh, UK, and the University of Sao Paulo, Brazil, by their local clinician. The research studies were approved by the Multicentre Research Ethics Committee for Scotland (05/MRE00/74) and the Ethics Committee of Hospital das Clinicas da Faculdade de Mediciana da Universidade de Sao Paulo (37868114.3.0000.0068), respectively. Informed written consent was obtained from all participating families. Families provided written consent for the publication of clinical photographs.

### DNA sequencing and variant validation

Genomic DNA was extracted from peripheral blood by standard methods. Trio whole-genome sequencing for A.III.2 and A.III.7 was performed at Edinburgh Genomics using Illumina SeqLab, which integrates Illumina TruSeq library preparation, Illumina cBot2 cluster generation, Illumina HiSeqX sequencing, Hamilton Microlab STAR integrative automation, and Genologics Clarity LIMS X edition. Detailed methods for DNA QC, library preparation, and sequencing are in the Supplemental Material.

For bioinformatics analysis, demultiplexing was performed using bcl2fastq (2.17.1.14), allowing one mismatch when assigning reads to barcodes. Adapters were trimmed during the demultiplexing process. BCBio-Nextgen (0.9.7) was used to perform alignment, BAM file preparation, and variant detection. BCBio used bwa mem (0.7.13) to align the raw reads to the human genome (GRCh38 with alt, decoy, and HLA sequences), samblaster (0.1.22) was used to mark the duplicated fragments (Faust and Hall 2014), and the genome analysis toolkit (3.4-0-g7e26428) was used for indel realignment and base recalibration (McKenna et al. 2010). Genotype likelihoods for each sample were calculated using the GATK HaplotypeCaller, and the resulting GVCF files were called jointly using GATK's GenotypeGVCFs function. Variant quality score recalibration (VQSR) was performed as per GATK best practices (Van der Auwera et al. 2013), with a truth sensitivity threshold of 99.9%. Following variant calling, variant calls were annotated with Ensembl's variant effect predictor (McLaren et al. 2016) and filtered to identify rare (AF < 0.5%) functional (nonsynonymous, splice site, and coding indels) variants consistent with biallelic inheritance in both sequenced individuals.

Whole-exome sequencing was performed on B.II.1 according to previously published protocols (Homma et al. 2019). Briefly, the library was constructed with SureSelect human all exon v7 kit (Agilent Technologies) according to the manufacturer's instructions. The exome library was sequenced on the NovaSeq platform (Illumina) running on paired-end mode. Reads were aligned to the GRCh37/hg19 assembly of the human genome. Variant calling was performed with Freebayes (Garrison and Marth 2012), and the resulting VCF was analyzed through the Franklin Genoox platform. Based on the family pedigrees indicating consanguinity, the exome data were screened for homozygous variants in the index patient, in addition to being absent in the gnomAD (Chen et al. 2024), ABraoM (Naslavsky et al. 2017), and SELAdb (Lerario et al. 2020) public databases (the last two being representative of the Brazilian population). Synonymous mutations were excluded. Data screening for deleterious variants was performed as reported previously (Homma et al. 2019). Candidate variants were submitted to the GeneMatcher platform (Sobreira et al. 2015) in search of additional cases with concordant genotype and phenotype. Variant interpretation followed the American College of Medical Genetics and Association for Molecular Pathology (ACMG-AMP) variant pathogenicity guidelines (Richards et al. 2015). Assessment of gene function was performed using the Online Mendelian Inheritance in Man (OMIM) and PubMed databases.

### Sanger sequencing

Variants were confirmed by bidirectional capillary dye terminator sequencing and annotated using the reference sequence (GenBank: NM_000075.4). Capillary sequencing was performed at the MRC Human Genetics Unit in Edinburgh, UK, and the University of Sao Paulo, Brazil. Primer sequences and PCR conditions for targeted *CDK4* sequencing are available on request.

### Splice site analysis

Variant determination was carried out using Alamut Visual Plus v.1.8 (SOPHiA Genetics), which used five distinct splice site prediction algorithms: SpliceSiteFinder-like, MaxEntScan, NNSPLICE, GeneSplicer, and Human Splicing Finder. Splice AI predictions were generated using the web application (Jaganathan et al. 2019).

### Cells and cell culture

Primary dermal fibroblasts were established from skin punch biopsies and maintained in AmnioMAX medium (Thermo Fisher Scientific 17001074) in 5% CO_2_ and 3% O_2_.

### Transcript analysis by RT-PCR

Cell pellets were harvested from control and patient-derived fibroblast cell lines; RNA was extracted using the RNeasy kit (Qiagen 74004), and cDNA was generated using the SuperScript III first strand synthesis system (Invitrogen 18080051). PCR-amplified products using 5′ and 3′ UTR primers and Phusion Flash high-fidelity PCR master mix (Thermo Scientific F548S) were isolated from 1% agarose gels, and DNA was extracted using a gel QIAquick gel extraction kit (Qiagen 28704). Products were cloned into a Topo vector using a Zero Blunt TOPO PCR cloning kit (Invitrogen 450245). Colony DNA was obtained using a QIAprep Spin minipreparation kit (Qiagen 27104), and colony PCRs were carried out using DreamTaq Green PCR master mix (Thermo Scientific K1081) and M13 forward and reverse primers for sequencing.

### Primers for RT-PCR

Primers used for RT-PCR were forward: GGTCTCCCTTGATCTGAGA**ATG** (22 bp long, 62°C Tm, and 50% GC) and reverse: TCAGTGTCCAGAAGGGAAATG (21 bp long, 62°C Tm, and 47.6% GC). The transcription start site is indicated in bold, and the 3′ end of the reverse primer is located 37 bp downstream from the CDK4 stop codon (ENST00000257904.11).

### RT-qPCR analysis of CDK4 splice variants and E2F target gene expression

Cell pellets were harvested from two control and patient-derived fibroblast cell lines at three independent times. RNA was extracted using an RNeasy Plus minikit (Qiagen 74134). cDNA was generated using a SuperScript III first strand synthesis system (Invitrogen 18080051) using random hexamer primers. CDK4 variant expression was assessed using SYBR Select master mix (Applied Biosystems 4472908) according to the manufacturer's recommendations (with *T*_a_ = 58°C). Primers spanning exons 5–7 were designed to amplify all potential CDK4 variants (CDK4-ctrl-FW: CGAAAGCCTCTCTTCTGTGGAAAC and CDK4-ctrl-RV: CAGGGATACATCTCGAGGCCAG). Primers spanning the exon 2/3 boundary were designed to monitor the presence of full-length CDK4 transcripts in P1 (CDK4-exon2/3-FW: ACTGAGGCGACTGGAGGC and CDK4-exon2/3-RV: GGTGCCTTGTCCAGATATGTCC). To monitor the truncated transcript in P1, primers were designed targeting the c.109-218del region (CDK4-c.109-218del-FW: CCTCAAGAGTGCTGATGGACG and CDK4-c.109-218del-RV: GGTGCCTTGTCCAGATATGTCC). Primers spanning the exon 3/4 boundary were designed to monitor the presence of full-length CDK4 transcripts in P2 (CDK4-exon3/4-FW: CCGAAACGATCAAGGATCTGATGC and CDK4-exon3/4-RV: CCAAAGTCAGCCAGCTTGACTG). To monitor the truncated transcript in P2, primers were designed targeting the c.354-521del region (CDK4-c.354-521del-FW: AGCCGAAACGATCAAGGTTGTTAC and CDK4-c.354-521del-RV: TTCGACGAAACATCTCTGCAAAGATAC). CDK4 complementation was measured using primers targeting a codon-optimized CDK4 sequence (CDK4-codonopt-FW#1: CCGCACGGATCGAGAAATTAAAG, CDK4-codonopt-RV#1: GGAGAAAGTCCAGACCTCGTAAG, CDK4-codonopt-FW#2: GGCAATAGTGAGGCGGATCAAC, and CDK4-codonopt-RV#2: CCATTTCGGGCACTACAGATTGTAC). E2F target gene expression was monitored using primers targeting to CDC6 (CDC6-FW#1: CCACTGTCTGAATGTAAATCACCTTC, CDC6-RV#1: AAGAGGGAAGGAATCTTGTGCTC, CDC6-FW#2: CTCTGGGGAAGTTATATGAAGCCTAC, and CDC6-RV#2: TCCAAGAGCCCTGAAAGTGAC) and PCNA (PCNA-FW: GCGTGAACCTCACCAGTATGT and PCNA-RV: TCCTGGTTTGGTGCTTCAAATACTAG). All reactions were normalized to the GAPDH housekeeping gene (GAPDH-FW: CGGATTTGGTCGTATTGGG and GAPDH-RV: TGGGTGGAATCATATTGGAAC).

### CDK4 and CDK6 complementation

Patient fibroblasts were transduced with lentiviral particles containing pLIX_403-CDK4 and/or pLIX_403-CDK6, a construct where full-length codon-optimized CDK4 or CDK6 was gateway-cloned into pLIX_403 (Addgene 41395 and 158560). The human CDK4 or CDK6 clones were obtained as pENTRY vectors from Twist Bioscience. CDK4-containing cells were selected with 0.5 µg/mL puromycin (Gibco A11138-03), and CDK6-containing cells were selected with 3.3 µg/mL blasticidin (Invivogen ant-bl-1).

### CDK6 RNAi

Dharmacon siGENOME smartpool against Ctl (nontargeting sequences) or human CDK6 (M-003240-02-0005) was used according to the manufacturer's instructions. Cells were transfected in 10 or 60 cm plates for 48 h before trypsinization and subsequently seeded for 24 h for quantitative microscopy as explained below.

### Protein modeling

For Supplemental Figure S1D, the AlphaFold model of human CDK4 (UniProt P11802) was visualized with PyMOL. The crystal structure of CDK4 in complex with Cyclin D3 (PDB 3G33) (Takaki et al. 2009) was visualized using UCSF ChimeraX (Meng et al. 2023).

### Immunoblotting

Total cell extracts were prepared in urea lysis buffer containing 8 M urea, 50 mM Tris-HCl (pH 7.5), 150 mM β-mercaptoethanol, protease inhibitors, and PhoSTOP (Roche 04693132001 and 4906837001). Lysed samples were sonicated seven times for 30 sec on/off cycles using a Bioruptor (Diagenode). Protein electrophoresis was performed using 10% NuPAGE or 4%–12% Bis-Tris mini protein gels (Invitrogen NP0336BOX and NP0301BOX) and MOPS running buffer (Invitrogen NP0001) at 80–130 V. Wet transfer of proteins to Immobilon-FL PVDF membrane (Millipore IPFL00010) was performed at 100 V for 60–75 min at 4°C. After transfer, membranes were washed in methanol, air-dried, reactivated in methanol, washed in 1× Tris-buffered saline/0.2% Tween-20 (TBS-T; Sigma P1379), and blocked in TBS-T/2.5% BSA (Roche 10735086001, lot 64758420) for 1 h at room temperature. Blots were incubated overnight in TBS-T/2.5% BSA containing primary antibody. After four 5 min washes in TBS-T, blots were incubated with secondary antibodies (1:20,000–1:30,000) for 1 h at room temperature, washed four times for 5 min in TBS-T, and rinsed in TBS before acquisition using a LI-COR Odyssey CLx imager. ImageStudio software was used for quantification.

The primary antibodies used in this study were as follows: CDK4 (Cell Signaling Technology 12790, RRID: AB_2631166): clone D9G3E, rabbit monoclonal to C-terminal CDK4; CDK4 (Proteintech 66950-1-Ig, RRID: AB_2882273): mouse monoclonal to full-length CDK4 (1:10,000; fusion prot. Ag20538); mouse anti-α-tubulin (α-Tub), clone B-5-1-2 (1:2000; Sigma T6074, RRID: AB_477582: lot 037M4804V); anti-pRB ser807/811 (Cell Signaling Technology 9308, RRID: AB_331472): rabbit polyclonal; anti-Cyclin D1 [SP4] (1:2500; Abcam ab16663); and anti-CDK6 (1:2500; Proteintech 14052-1-AP).

Secondary antibodies used for immunoblotting in this study were as follows: 0.1 mg of IRDye 680RD goat antirabbit IgG (H + L) highly cross-adsorbed (LI-COR Biosciences 925-68071, RRID: AB_2721181), 0.1 mg of IRDye 800CW goat antimouse IgG (H + L) highly cross-adsorbed (LI-COR Biosciences 925-32210 [also 925-32210], RRID: AB_2687825), 0.1 mg of IRDye 680RD goat antimouse IgG (H + L) highly cross-adsorbed (LI-COR Biosciences 925-68070, RRID: AB_2651128), 0.1 mg of IRDye 800CW goat antirabbit IgG (H + L) highly cross-adsorbed (LI-COR Biosciences 925-32211, RRID: AB_2651127), and 0.1 mg of IRDye 680RD donkey antimouse IgG (H + L) highly cross-adsorbed (LI-COR Biosciences 925-68072, RRID:AB_2814912).

### Growth curve

Human primary fibroblasts (1.5 × 10^5^ cells) were seeded on day 0 into a T25 flask in 3% O_2_ and split and counted every 3 days, and 1.5 × 10^5^ cells were reseeded into a new flask. Counts were measured in duplicate using a Countess automated cell counter according to the manufacturer's instructions. Doubling times were calculated during log phase growth (days 3–15) using the formula: *t*/log_2_ (*e*/b), where *t* = time in hours, *e* = final population size, and *b* = population size at the start of log phase growth.

### Flow cytometry

#### BrdU incorporation

Exponentially growing cells were pulsed with 64 µM BrdU for 40 min (in prewarmed media), rinsed once with PBS, trypsinized, pelleted at 1200*g*, resuspended in 75 µL of PBS, fixed by adding 1 mL of 100% freezer-cold ethanol with gentle vortexing, and stored at −20°C. Fixed cells were pelleted by centrifugation at 1300*g* for 5 min and then washed in 1× PBS/0.1% Triton-100-X (PBS-T).

DNA denaturation was performed in 15 mL Falcon tubes with all centrifuge steps at 300*g*, as described previously (Darzynkiewicz and Juan 2001). Cells were centrifuged at 300*g* for 10 min, resuspended in 1× PBS-T, and centrifuged for 6 min. The pellet was resuspended in 1× PBS-T/0.1 M HCl, incubated for 2 min at room temperature, and then centrifuged. The pellet was resuspended in 1.5 mL of DNA denaturation buffer (0.15 mM NaCl, 15 µM trisodium citrate dihydrate), heated for 5 min at 95°C, and then chilled immediately on ice. Five milliliters of antibody diluting buffer (ADB; 1× PBS, 0.1% Triton, 1% FBS) was added, and the cells were centrifuged.

For staining, cells were transferred to 1.5 mL tubes with centrifuge steps at 1200*g*. The pellet was resuspended in 50 µL of ADB plus rat anti-BrdU (1:600; Abcam ab6326), incubated for 60 min at room temperature, washed in 1 mL of ADB, and centrifuged for 5 min. Cells were incubated in ADB plus goat antirat Alexa fluor 488 secondary antibody (1:1500; Invitrogen A11006) for 45 min at 4°C, washed in 1 mL of ADB, and centrifuged for 5 min. The cells were incubated in 1 mL of ADB containing DAPI (1:1000; final concentration of 20 µg/mL) for 5 min at room temperature, centrifuged, and resuspended in 350 µL of PBS.

A Cytoflex S analyzer (Beckman Coulter) was used with the violet 405 nm laser and 450/45 bandpass filters for DAPI detection and the 450 nm laser and 525/50 filter for BrdU detection. Ten-thousand to 20,000 events in the single-cell population gate were recorded. Data analysis was performed using FlowJo v10.8.1 (FlowJo LLC, BD).

#### p-Histone H3 ser10

Pellets of exponentially growing cells were suspended in 500 µL of 2% paraformaldehyde (PFA) in PBS and fixed for 15 min on ice. Five-hundred microliters of PBS/0.1% Triton was added and cells were spun down at 1000*g* and resuspended in 500 µL of FACS storage buffer (3% hi FBS/PBS/0.09% Na-azide) for several days at 4°C. Cells were stained with mouse anti-p-Histone H3 ser10 (1:300; Cell Signaling Technology 9706 GG3) using the same staining procedures as shown above for flow cytometry staining.

#### Immunofluorescence

Cells were seeded in glass-bottom 8 well chambers (Ibidi 80807) and fixed for 5 min at room temperature in Psuc (4% PFA, 2% sucrose, PBS), followed by methanol:acetone (1:1) for 3 min at −80°C. To block, cells were then washed three times for 5 min in PBS and incubated for 1 h at room temperature in block solution (1% BSA/PBS or 10% goat serum + 0.1% triton/PBS). To stain, cells were incubated with primary antibodies in block solution overnight at 4°C, rinsed three times for 5 min with wash solution (1% BSA/PBS or 0.5% goat serum + 0.1% triton/PBS for fibroblast cdk4 staining), incubated with secondary antibody in wash solution for 1 h at room temperature, and washed three times for 5 min. To stain DNA, cells were incubated with DAPI in 1 mg/mL PBS (1:2000) for 5 min at room temperature and then washed three times for 5 min in PBS. Cells were stored in PBS until imaging.

For pericentrin/α-tubulin staining, we used the primary antibodies rabbit antipericentrin (1:400; Abcam ab4448) and rat anti-α-tubulin (1:500; Serotec MCA77G) and secondary antibodies 568 goat antirabbit (1:500; Invitrogen A11036) and 488 goat antirat (1:500; Invitrogen A11006).

For Cdk4/α-tubulin staining in fibroblasts, we used the primary antibodies rabbit anti-Cdk4 (1:1000; Cell Signaling 12790) and rat anti-α-tubulin (1:1000; Serotec MCA77G) and secondary antibodies 647 goat antirabbit (1:2000; Invitrogen A21244) and 488 goat antirat (1:200; Invitrogen A11006).

#### p-RB ser807/811 and total RB staining in fibroblasts

Cells were seeded in glass-bottom 8 well chambers (Ibidi 80807) using 7500 cells/well and cultured for 24–72 h. Doxycycline (0.4 µg/mL) and/or 5 mg of 0.2 µM CDK4/6 inhibitor palbociclib (Cambridge Bioscience HY-50767) or DMSO was added and cells were grown for 24 h before a 25 min 40 µM EdU (Sigma 9000584) pulse before cell fixation in 2% PFA/PBS for 20 min at 4°C. Cells were washed three times for 5 min in PBS, permeabilized in 0.3% Triton/PBS for 15 min, and incubated for 1 h at room temperature in block solution (5% goat serum/0.1% Triton/PBS). Cells were then incubated with microscopy-validated antibodies to p-RB ser807/811 (Cell Signaling Technology 8516D2B12 XP) and total RB (Cell Signaling Technology 9309 4H1) at 1:2000 dilution in block solution overnight at 4°C, rinsed three times for 5 min in 0.1% Triton/PBS, and incubated with secondary antibodies AF568 goat antirabbit (Invitrogen A11036) and AF488 goat antimouse (Invitrogen A11029) at 1:3000 in block solution for 1 h at room temperature. After a 5 min wash, cells were incubated for 30 min at room temperature in EdU labeling buffer, adding the following components in order (for 1 mL): 100 µL of 20 mM CuSO_4_, 100 µL of 0.5 M L-ascorbic acid, 800 µL of 0.1%Triton/PBS, and 0.2 µL of Alexa fluor 647 Azide. Three 5 min washes were followed by nuclear staining using DAPI, as shown above.

#### S-phase time by microscopy

A full protocol for S-phase time measurements shown in Supplemental Figure S4B was presented previously (Parry et al. 2020); this time, a 64 μM BrdU pulse for 2.25 h was followed by a 15 min 40 μM EdU pulse. Cells were then fixed in 4% PFA. DNA was denatured with 1× PBS/0.1% Triton/0.1 M HCl for 15 min and washed, and BrdU was detected using mouse anti-BrdU MoBu-1 (Abcam ab8039) at 1:600 dilution overnight at 4°C. The following day, AF488 goat antimouse secondary antibody (1:3000) was used to detect BrdU for 1 h at room temperature. The click reaction using AF647-azide to detect EdU was followed as shown above for QIBC studies.

### Image analysis

Mitosis detection and pericentrin spot imaging were carried out using ScanR (Olympus). Detailed methods for wide-field imaging and image analysis are in the Supplemental Material (van Rossum 1995; McKinney 2010; Schindelin et al. 2012; Harris et al. 2020; R Core Team 2022).

### ScanR QIBC pRB and total RB

Chamber slides were automatically imaged in ScanR wide-field mode using a UPLXAPO 40× 0.95 NA objective (Olympus) using a Lumencor SpectraX LED light source (Lumencor) and Semrock Briteline DAPI/FITC/Cy3/Cy5 optical filters to detect DAPI, total RB, pRB-ser807/811, and EdU. For further details on the wide-field imaging acquisition, see the Supplemental Material. Seventy-two images per well averaging 1500–2000 cells per condition were acquired and analyzed for each independent experiment using the ScanR acquisition software (Olympus). After background correction, nuclei were segmented using the artificial intelligence module for object identification, and the fluorescence intensity of each channel was generated for each object. Single cells were gated by a nuclear area/circularity gate-excluded doublets, and a subsequent DNA content histogram gate using DAPI selected the single-cell population. Subsequent DAPI versus EdU plots were used to gate cell cycle stages. G0/G1, S, and G2/M were as as documented in Figure 6A. pRB and RB levels were quantified for gated cell cycle populations and are depicted in Figure 6 and Supplemental Figure S6. Here, the ScanR analysis software was used to generate fluorescence intensity values per object and for each individual well and gate. The total and fraction of cells in each gate were exported from the ScanR analysis. Tableau software (license for researchers; https://public.tableau.com/app/discover) was used to generate the plots presented in Figure 6.

### Statistical analysis

Statistical testing was performed using GraphPad Prism v.10. Two-sided parametric (*t*-tests), one-sample *t*-tests, or nonparametric Mann–Whitney *U*-tests were performed for quantitative measurements as indicated in the figure legends. A one-way ANOVA test was performed for cell doubling time; significance (*P*-values) is indicated in the figures or legends. The number of samples and/or experimental replicates is indicated in the figures and legends.

### Data availability

WGS data will be deposited at the European Genome Archive on publication for individuals from family A. WES data for family B are not consented for deposition.

## Supplemental Material

Supplement 1
